# Irregularities and Their Corrections

**Published:** 1888-09

**Authors:** W. E. Magill

**Affiliations:** Erie, Pa.


### IRREGULARITIES AND THEIR CORRECTION.

BY W. E. MAGILL, D. D. S., ERIE, PA.

READ BEFORE THE PENNSYLVANIA STATE SOCIETY, JUNE 7, 1888.

When irregular teeth are presented for examination and advice, the first question to decide is, whether you had not better let them alone. This is the question of questions, upon the wise decision of which may depend your reputation, your own comfort for a considerable period; and not only the comfort, but also various important influences in the life of your patient.

No hasty conclusions should be adopted. Nature can do wonders, and may be competent, if given time, to right all that seems wrong.

To one who feels able to cope with mechanical difficulties, this department of practice is fascinating; and the greater the difficulties presented, the more absorbing becomes the interest of the operator. To bring order out of chaos, to introduce symmetry where distortion has prevailed, to establish beauty in the place of homeliness, is no mean ambition; and the man who succeeds in so doing may well be called and possess the true spirit of a reformer.

When satisfied that no intervention is needed, it then becomes your duty to make plain as possible to the patient the grounds of your decision, the reasons for your opinion. This is very important when you advise delay, for a little dissatisfaction with your course, coupled with impatient desire to be good-looking, may send your patient to Dr. Forceps, who is always ready to advise and take the short-cut of extraction, because it brings the certainty of cash in hand.

When you have decided that interference is necessary, you have reached the foot of the hill "Difficulty" only. The full responsibility of a careful diagnosis is upon you, and "to pull or not to pull" is often a very important question. Irregularity usually presupposes want of room, and continued want of room is a barrier to success in any attempt to regulate. My own experience tells me that at this critical time we are likely to fail for want of a thorough examination and consideration of each case in all its bearings. We may undertake to spread an arch when better articulation could be more easily secured by extraction, and all other benefits as surely gained or retained, as they could by long and laborious—perhaps painful—interference.

It is possible for us to start out with an exaggerated estimate of the value of the individual natural tooth. This is all right when viewed from the side of conservative dentistry, or when a comparison is drawn between the natural organ and an artificial substitute; but it is out of place when interfering with a proper procedure to secure the greatest good to the greatest number. As in civilized society, the interests of the individual give way when they contravene the public good, so the value of a single tooth is more than counterbalanced by the importance which attaches to the other members of the arch.

We are under obligations to take the best, the most direct, and least painful plan. If that involves the extraction of a tooth, we should have no hesitation about its removal. The attempt to force teeth into positions where room has not been provided, risks failure, protracted efforts and pain to the patient, without the encouragement of success. Sometimes, too, this mistaken economy results in successful enlargement of the arch, but away from proper antagonism to the opposing arch, and outside the line of beauty for the mouth. Then there is the ever-present tendency to return, always more powerful where abundant room is not secured; the necessity of wearing a retaining device for a long time, and sometimes the annoying experience of seeing, ultimately, the teeth in their old position.

In this early diagnosis the condition of the alveolar arch is a very important consideration. If it is in good form and width as compared with the opposing arch, and if, in the main, articulation is good, the irregularity consisting in the deviation of a few teeth from their proper position, the probability is that judicious extraction is the short and direct road to success. Not necessarily the removal of all that are out of line, perhaps none of those that are irregular. Sometimes irregularity of the bicuspids, or even of the cuspids, may be satisfactorily remedied by the removal of a defective molar. This plan may require the additional use of appliances, but be justified by the exchange of a diseased tooth for one that is sound. It has become a settled and, I think, a wise practice, to preserve the cuspids, as more important, and extract one of the bicuspids, in those common cases where, all the teeth being sound, the cuspids have developed outside the arch. Some operators, however, do not hesitate to extract the cuspids. If this is done as the result of ignorance, such ignorance is culpable—the practice is certainly in violation of good taste.

The literature of our profession proves that experience has given us established rules of practice, some of which have crystallized into principles for general application. I refer with pleasure to the work of Dr. Kingsley, entitled "Oral Deformities," and to the more recent publication, "American System of Dentistry," and what is there presented under the title "Orthodontia."

It is generally conceded to be good practice to postpone serious and extensive interference, with a view to regulate teeth, until the complete development of the permanent set, save the third molars. I think we may adopt the rule that no considerable force should be applied to a partly-developed tooth to drive it into proper position.

In the case of upper teeth developing inside the lower arch, I would fit a plate with biting blocks, to give room; and rely upon mere guidance or impingement upon the palatine surface of the coming tooth, leaving to nature the application of force.

I have been much interested in irregularities caused by disease of the investing membrane. This usually consists in a movement to the right or left, in cases involving front teeth ; but the movement of molars is usually in the line of pressure from occlusion. Incisors sometimes elongate, and sometimes the side movement continues to the extent of overlapping a neighboring tooth. In these cases mechanical appliances may be used as aids, the true corrective being treatment of disease in the tooth socket.

Judgment is shown in the selection, and skill in the successful application of the appliance selected. I have but little to say of appliances, however, because our professional museum is so well stocked that American ingenuity would deserve high rank if judged alone by devices to regulate teeth.

It has been reported of Archimedes that he said he could, with his lever, move the world if only a sufficient fulcrum could be furnished. The dentist, too, has serious thoughts about a fulcrum when he is trying to move a bicuspid towards a molar and finds the accommodating molar rapidly approaching the bicuspid, while the latter remains in situ. For such cases I think we yet need a pulley and tackle combination, or a device which shall give the short arm of a lever to the bicuspid and the long arm to the molar.

I am in favor of intermittent force in moving teeth, for the following reasons :

1st. It involves less pain for the patient, or, what is equivalent, gives periods of rest and relief from soreness.

2d. It is the safe plan, for it is but seldom that we can move a tooth without some irritation of the investing membrane. If we limit movement we may limit irritation, and therefore have less anxiety about the case when the patient is beyond our reach.

3d. It gives time for the natural process of repair. It is of no value to move a tooth so rapidly that in its wake shall develop an open fissure.

Therefore, for power, for ease of control and convenience, preference should be given to the screw or wedge, or combinations of screw and wedge, or screw and lever.

